# Public Health Interventions’ Effect on Hospital Use in Patients With COVID-19: Comparative Study

**DOI:** 10.2196/25174

**Published:** 2020-12-23

**Authors:** Xiaofeng Wang, Rui Ren, Michael W Kattan, Lara Jehi, Zhenshun Cheng, Kuangnan Fang

**Affiliations:** 1 Department of Quantitative Health Sciences Cleveland Clinic Cleveland, OH United States; 2 Department of Statistics Xiamen University Xiamen China; 3 Neurological Institute Cleveland Clinic Cleveland, OH United States; 4 Department of Pulmonary and Critical Care Medicine Zhongnan Hospital of Wuhan University Wuhan China

**Keywords:** COVID-19, public health, intervention, hospital, use, prediction, comparative, United States, China, implementation, observational

## Abstract

**Background:**

Different states in the United States had different nonpharmaceutical public health interventions during the COVID-19 pandemic. The effects of those interventions on hospital use have not been systematically evaluated. The investigation could provide data-driven evidence to potentially improve the implementation of public health interventions in the future.

**Objective:**

We aim to study two representative areas in the United States and one area in China (New York State, Ohio State, and Hubei Province), and investigate the effects of their public health interventions by time periods according to key interventions.

**Methods:**

This observational study evaluated the numbers of infected, hospitalized, and death cases in New York and Ohio from March 16 through September 14, 2020, and Hubei from January 26 to March 31, 2020. We developed novel Bayesian generalized compartmental models. The clinical stages of COVID-19 were stratified in the models, and the effects of public health interventions were modeled through piecewise exponential functions. Time-dependent transmission rates and effective reproduction numbers were estimated. The associations of interventions and the numbers of required hospital and intensive care unit beds were studied.

**Results:**

The interventions of social distancing, home confinement, and wearing masks significantly decreased (in a Bayesian sense) the case incidence and reduced the demand for beds in all areas. Ohio’s transmission rates declined before the state’s “stay at home” order, which provided evidence that early intervention is important. Wearing masks was significantly associated with reducing the transmission rates after reopening, when comparing New York and Ohio. The centralized quarantine intervention in Hubei played a significant role in further preventing and controlling the disease in that area. The estimated rates that cured patients become susceptible in all areas were small (<0.0001), which indicates that they have little chance to get the infection again.

**Conclusions:**

The series of public health interventions in three areas were temporally associated with the burden of COVID-19–attributed hospital use. Social distancing and the use of face masks should continue to prevent the next peak of the pandemic.

## Introduction

A novel coronavirus was identified as the cause of a cluster of pneumonia cases in Wuhan, the capital city of Hubei Province, China, at the end of 2019. It quickly spread throughout China, followed by an increasing number of cases in other countries. The virus was named SARS-CoV-2. The World Health Organization (WHO) designated the disease caused by SARS-CoV-2 as COVID-19. The disease’s main clinical manifestations include fever, cough, shortness of breath, fatigue, and dyspnea [1-4], with progression to multi-organ dysfunction in severe cases. In March 2020, the WHO declared the COVID-19 outbreak a global pandemic. As of October 20, 2020, there are 40,756,188 cases (including 1,124,627 deaths) attributed to COVID-19 that have been reported worldwide, with sustained transmission in many countries, including the United States.

Many statistical and mathematical models were proposed to estimate the dynamics and the potential spread of COVID-19 [5-9]. The classical compartmental models such as the susceptible-exposed-infectious-removed (SEIR) models were the most widely used [10]. These models were applied to estimate the transmission risks, predict the numbers of infected subjects, and evaluate the public health interventions [9,11,12]. A key parameter in these models was the basic reproduction number (R_0_), defined as the expected number of additional cases that one case will generate. If R_0_ is above 1, continuous human-to-human transmission with sustained transmission chains will occur. The median R_0_ of COVID-19 was recently estimated [12] at 2.79 (IQR 1.16), which is significantly higher than that of the severe acute respiratory syndrome coronavirus.

Admissions to hospitals and intensive care units (ICUs) increased exponentially during the first few weeks of the outbreak in many countries, significantly straining resources and, at times, transforming a public health emergency into an operational crisis [13]. Rigorous government control policies were instituted to slow the spread of the disease and reduce the burden of COVID-19–attributed hospital use. Although associations of public health interventions with COVID-19 epidemiology in Wuhan City were studied [14,15], the effects of these interventions on hospital use at a state level remain to be investigated. This knowledge gap is significant given the growing public health concern regarding the adequacy of resources to treat severe COVID-19, including hospital beds, ICUs, and ventilators, in the United States. To project the timing of the outbreak peak and the number of ICU beds required at the peak, Moghadas et al [11] simulated a COVID-19 outbreak parameterized with the US population demographics. Grasselli et al [16] presented a linear model as well as an exponential model to predict ICU admissions.

However, most of the current models used prespecified parameters from the literature to simulate the COVID-19 outbreak [17]. They did not account for dynamic disease evolution, which often resulted in underestimating or overestimating hospital use. In this study, we propose a novel generalized dynamic SEIR model. The clinical stages of COVID-19 are stratified in the model, and the effects of public health interventions are modeled through piecewise exponential functions. Unlike the other existing methods, we estimated all dynamic parameters from observed epidemic data through Bayesian inferences.

In the United States, New York State was hit the hardest by COVID-19 in the early stage of the pandemic, and Ohio State had early public health interventions by its government and medical community. In China, Hubei Province was where COVID-19 was first detected, and the province was put under strict lockdown. Using the proposed model, we aim to evaluate and compare the effectiveness of public health interventions on hospital use in patients with COVID-19 for the three representative areas; in addition, we aim to study the time-dependent associations between the interventions and transmission rates and effective reproduction numbers.

## Methods

### Data Sources

The epidemiological data of COVID-19 in New York State and Ohio State were obtained from the Centers for Disease Control and Prevention of the United States, Johns Hopkins Coronavirus Resource Center [18], and the COVID tracking project from the Atlantic [19]. We collected the daily number of confirmed cases, the number of cumulative deaths, and the number of cumulative cured cases from March 12 to September 14, 2020. In addition, we obtained the number of hospitalization cases from March 21 to September 14, 2020, in New York and the numbers of hospitalized, mild, severe, and critically ill patients in Ohio from May 2 to September 14. In this study, the data from March 12 to August 31 were used for model building, and the data from September 1 to September 14 were used for external validation.

The epidemiological data of COVID-19 in Hubei, China were mainly obtained from the National Health Commission of China, Chinese Center for Disease Control and Prevention, and Hubei Provincial Health Commission [20]. We collected the daily numbers of confirmed infected cases, cured cases, and deaths from December 1, 2019, to March 31, 2020. We also extracted the numbers of hospitalized, mild, severe, and critically ill patients from January 26 to March 31, 2020.

### Dynamic SEIR Model

In the classical SEIR model, the human-to-human transmission of COVID-19 was modeled using a compartmental representation of the disease where an individual occupied one of the four states: susceptible (*S*), exposed (*E*), infectious (*I*), and removed (*R*). The population was assumed to have a homogeneous spatial distribution. Susceptible individuals could acquire the virus through contact with individuals in the infectious compartment and become exposed. The exposed individuals were infected but not yet infectious. They experienced an incubation duration and progress to the infectious stage at a certain rate. The infectious individuals could progress into the removed stage (usually recovered with immunity) at another rate.

To characterize the hospital use for patients with COVID-19, we generalized the classical SEIR model by introducing a few new compartments. We considered three clinical stages for COVID-19 in our model [4,21]: mild, severe, and critical. Mild included patients who had symptoms like fever and cough, and may have mild pneumonia. Hospitalization was not required in the United States, but such individuals were required to admit to temporary hospitals in China. Severe was characterized by dyspnea, respiratory frequency≥30/minute, blood oxygen saturation≤93%, PaO2/FiO2 ratio<300, or lung infiltrates>50% within 24-48 hours. Hospitalization and supplemental oxygen were generally required for them. Critical cases exhibited respiratory failure, septic shock, or multiple organ dysfunction and failure. Treatment in an ICU, often with mechanical ventilation, was typically required.

**Figure 1:**
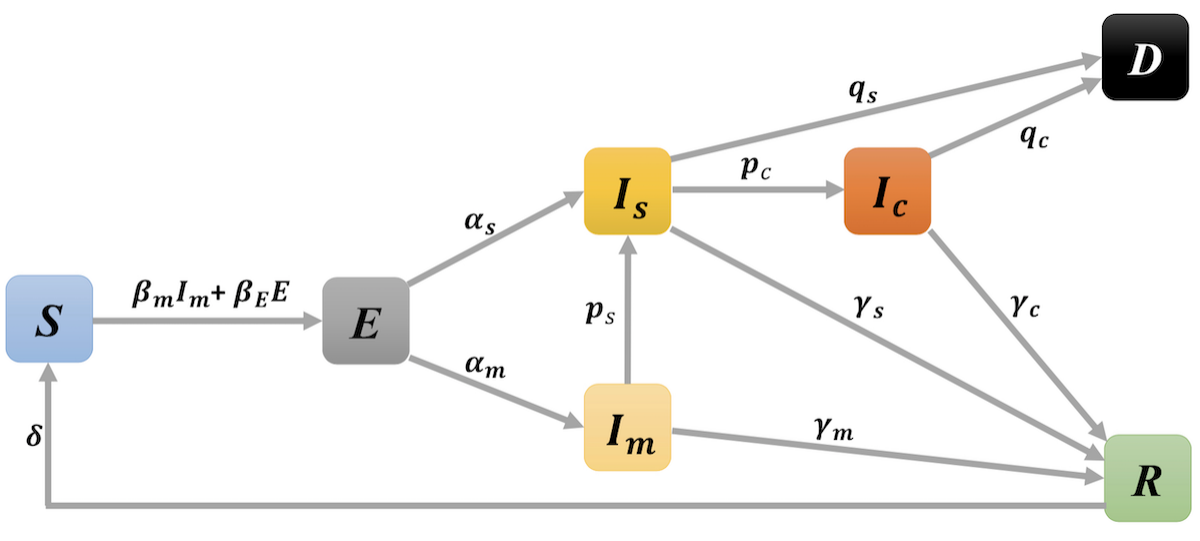
Flowchart of the dynamic susceptible-exposed-infectious-removed model.

Figure 1 displays the flowchart of the proposed model. Here, the infectious stage includes all individuals who are confirmed COVID-19 cases, which is composed of three subcompartments: *I_m_* represents the infectious individuals with mild symptoms, *I_s_* represents the severe patients, and *I_c_* represents the patients who were critically ill. The removed stage includes two subcompartments: the dead compartment (*D*) and the recovered compartment (*R*).

We assumed that an individual who is susceptible or at risk can only be infected by exposed individuals (patients with COVID-19 who had not been confirmed) or mild patients, since severe or critically ill patients were hospitalized and had a small (ignorable) probability to infect others. In Figure 1, *β_m_* is the transmission rate that infectious individuals in the compartment *I_m_* contact susceptibles and infect them, and *β_E_* is the transmission rate at which exposed individuals in *E* contact susceptibles and infect them. α_*_ is the rate of progression from the exposed to infectious class I_*_, where the subscript “_*_” denotes one of the patient groups, mild (m), severe (s), and critically ill (c). γ_*_ is the rate that infectious individuals in class I_*_ recover from the disease. *p_s_* is the rate that infectious individuals in *I_m_* progress to *I_s_*, and *p_c_* is the rate that infectious individuals in *I_s_* progress to *I_c_*. *q* represents the death rate for patients who are critically ill. Since the WHO recently stated that there was no evidence that patients who recover from COVID-19 are entirely immune, we used *δ* to represent the rate that infected individuals are cured and become susceptible again. The mathematical details of the proposed model are given in Multimedia Appendix 1.

Mathematically, our model is expressed as a system of ordinary differential equations:


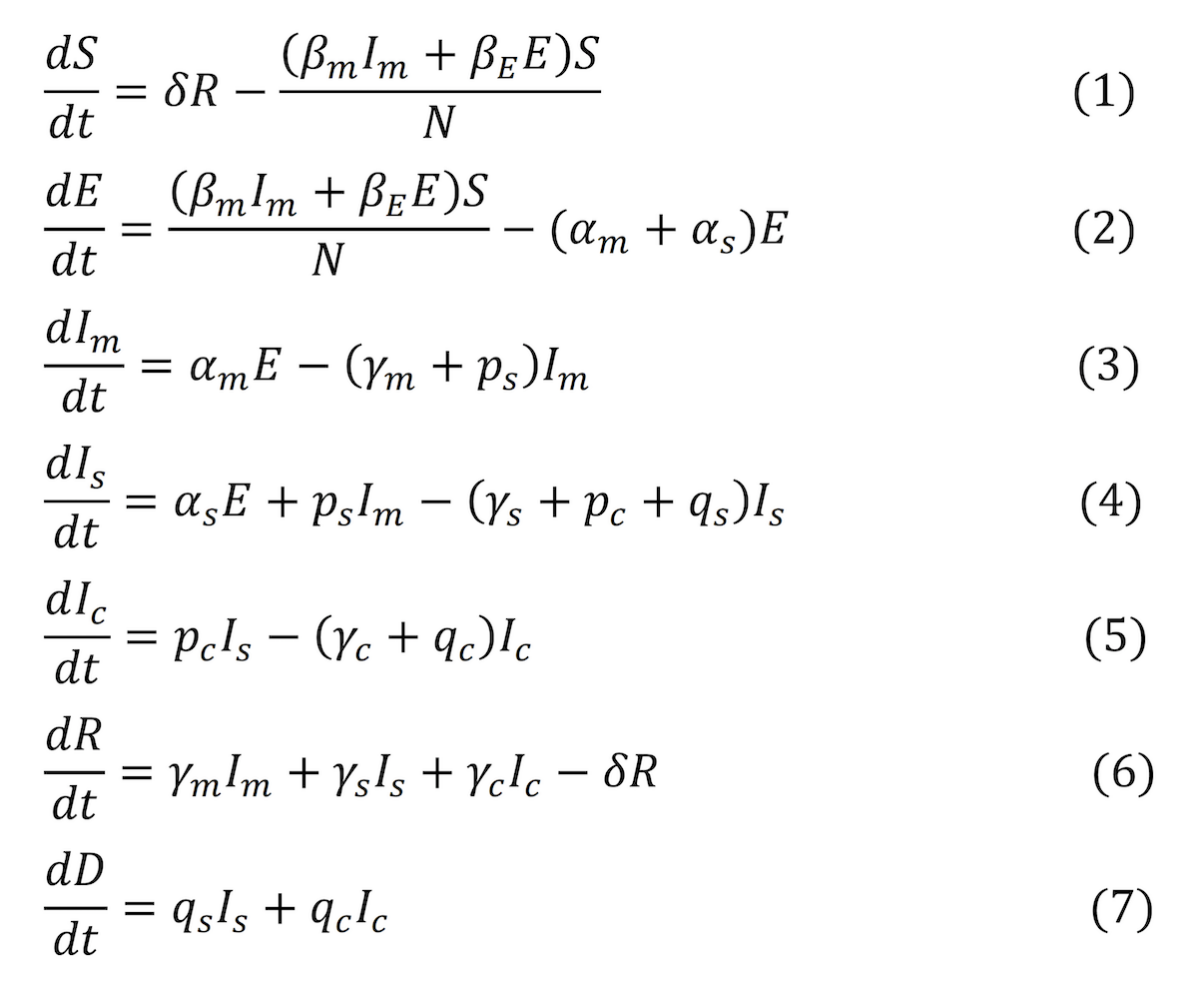


with the size of population *N*:

*N* = *S*(*t*) + *E*(*t*) + *I_m_*(*t*) + *I_s_*(*t*) + *I_c_*(*t*) + *R*(*t*) + *D*(*t*) **(8)**

### Public Health Interventions

#### Classification of Time Periods in New York

**Figure 2:**
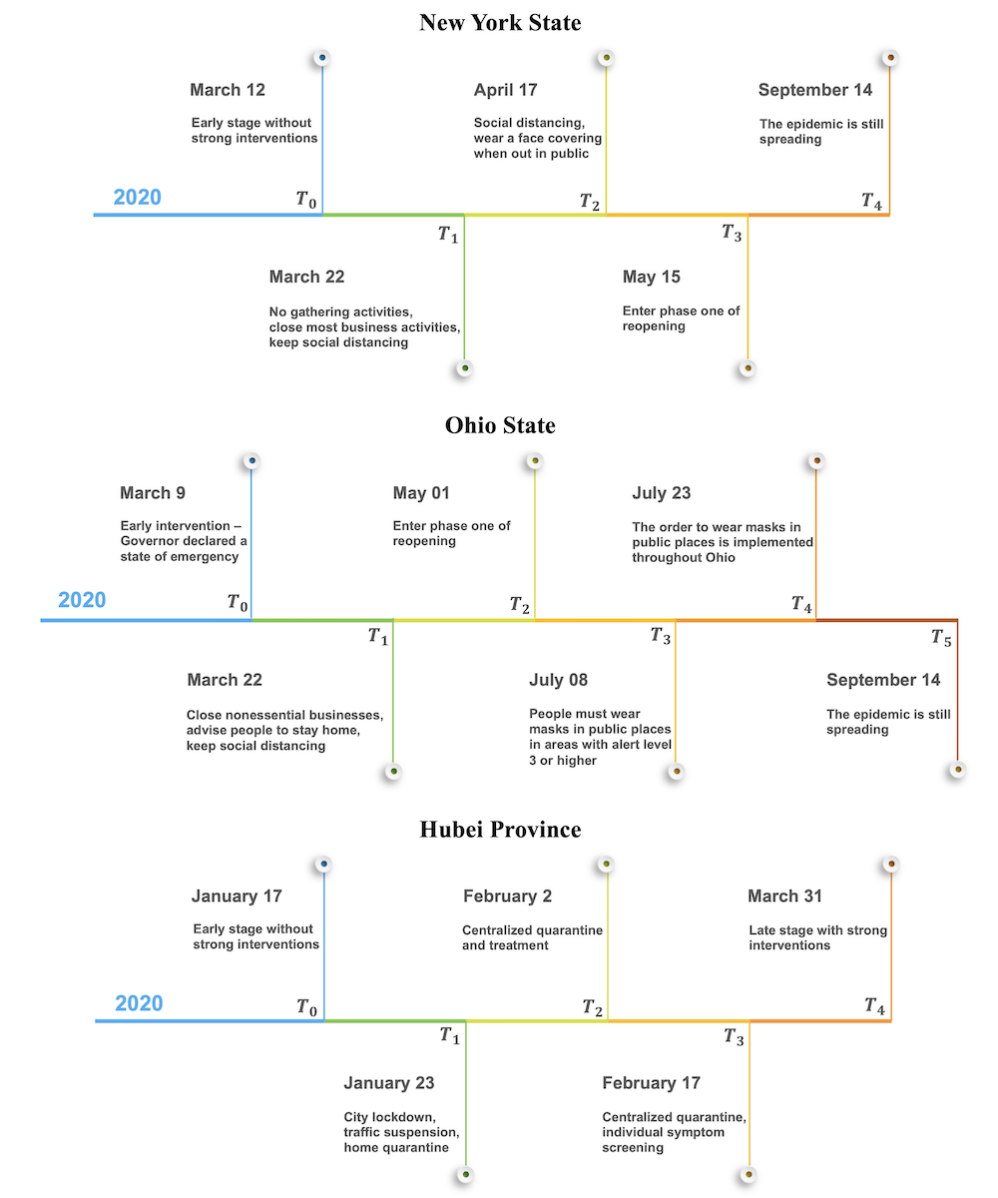
Classification of time periods according to key events and interventions in New York State, Ohio State, and Hubei Province.

To better reflect the epidemic trends and corresponding interventions of COVID-19, four periods were classified according to data relevant for virus transmission in New York State (Figure 2). The first period was from March 12 to 22, 2020, the free virus transmission period. The second period was from March 22 to April 17. The governor of New York State, Andrew Cuomo, issued the “New York State on PAUSE” (Policies Assure Uniform Safety for Everyone) Executive Order on March 20, which took effect on March 22. This 10-point policy included a new directive that all nonessential businesses statewide must close in-office personnel functions, temporary banning of all nonessential gatherings of individuals of any size for any reason, and mandated social distancing of at least 6 feet from others for individuals in public. The third period was from April 17 to May 15. The governor announced another order that all people in New York would be required to wear a face covering when out in public and in situations where social distancing cannot be maintained. The fourth period started on May 15. Some regions of New York State started to enter the first phase of reopening. To reflect the effects of governmental public health policy in the different periods, we modeled the transmission rates using piecewise exponential functions (see Multimedia Appendix 1).

#### Classification of Time Periods in Ohio

We considered five time periods based on several key time points that could affect the virus’s spread in Ohio. Ohio was the state that took early actions against COVID-19. The first period was from March 9 to 22, 2020. The governor of Ohio, Mike DeWine, declared a state of emergency in Ohio on March 9 and announced several steps to fight against the spread of the COVID-19 epidemic; before, there were no confirmed cases in Ohio. The second period was from March 22 to May 1. A statewide “stay at home” order was issued on March 22. The order included advising people to stay at home, closing most nonessential businesses, and requiring individuals in public to practice social distancing. The third period was from May 1 to July 8. Ohio entered the first phase of its reopening program. The fourth period was from July 8 to 23, 2020. Mr DeWine announced that any county at level 3 or higher in the Public Health Advisory System must wear masks in public places. The fifth period started from July 23. Ohio mandated the use of masks and face coverings across the state while in public.

#### Classification of Time Periods in Hubei Province

We considered four time periods according to the critical dates that may have affected virus transmission in Hubei. The first period was from January 17 to 23, 2020, the Spring Festival period in China. There was a large-scale population flow, and there was no strong intervention to prevent and control the epidemic. The second period was from January 23 to February 2. The China’s central government imposed a lockdown in Wuhan and other cities in Hubei to quarantine the center of the COVID-19 outbreak on January 23. The actions included suspending public transport, banning all vehicles in cities, blocking the main roads between cities, requiring people to wear masks in public places, and forbidding gathering activities. The third period was from February 2 to 17. Hubei successively opened several new temporary hospitals to treat patients with mild symptoms and isolate the infection source. The fourth period started on February 17. The government began the individual symptom screening for all residents.

#### The Effects of Medical Resources

In the early period of the COVID-19 pandemic, medical resources such as hospital beds, ICU beds, and ventilators fell short. During this period, the availability of the medical workforce was affected since many physicians and nurses were becoming ill or quarantined [22]. With the continuous allocation of more medical resources and the opening of new temporary hospitals, patients with mild disease got a better treatment. Therefore, we considered that the cure rate *γ_m_* changed with time and modeled it with a three-parameter logistic function to reflect the continuous improvement of reactive medical resources in the three areas.

### Statistical Analysis

We performed a Bayesian analysis with Markov chain Monte Carlo (MCMC) to fit the models to the COVID-19 epidemiological data in the three areas. To implement the MCMC algorithm, we followed the method by Raftery and Lewis [23]. After an initial number of 10,000 burn iterations, every 10th MCMC sample was retained from the next 200,000 samples. Thus, 20,000 samples of targeted posterior distributions were used to estimate the unknown parameters. The stability of the posterior distributions was checked by examining the graphics of the runs. The mathematical details of the dynamic SEIR model are presented in Multimedia Appendix 1. We also developed an online R shiny app to help readers assess the models [24]. All analyses were done with R software (V3.6.3; R Foundation for Statistical Computing).

## Results

### Parameters

The parameters in the dynamic models included the transmission rates *β_m_* and *β_E_*; the progression rates *α_m_*, *α_s_*, *p_s_*, and *p_c_*; the recovery rates *γ_m_*, *γ_s_*, and *γ_c_*; the death rates *q_s_* and *q_c_*; and the rate that recovered individuals become susceptible again, *δ*. Posterior means and 95% credible intervals (CIs) for the model parameters are presented in Table S1 in Multimedia Appendix 1. Those estimated parameters varied for the three areas. Noticeably, we found that the rate *δ* was low and ignorable in all areas, which indicates that the patients who were cured had little chance to become susceptible again. The fitted curves for the epidemic trends of COVID-19 for the three areas are presented in Figure S1 in Multimedia Appendix 1. The proposed models performed well in simultaneously fitting the multidimensional epidemic data.

### Transmission Rates and Effective Reproduction Numbers

**Figure 3:**
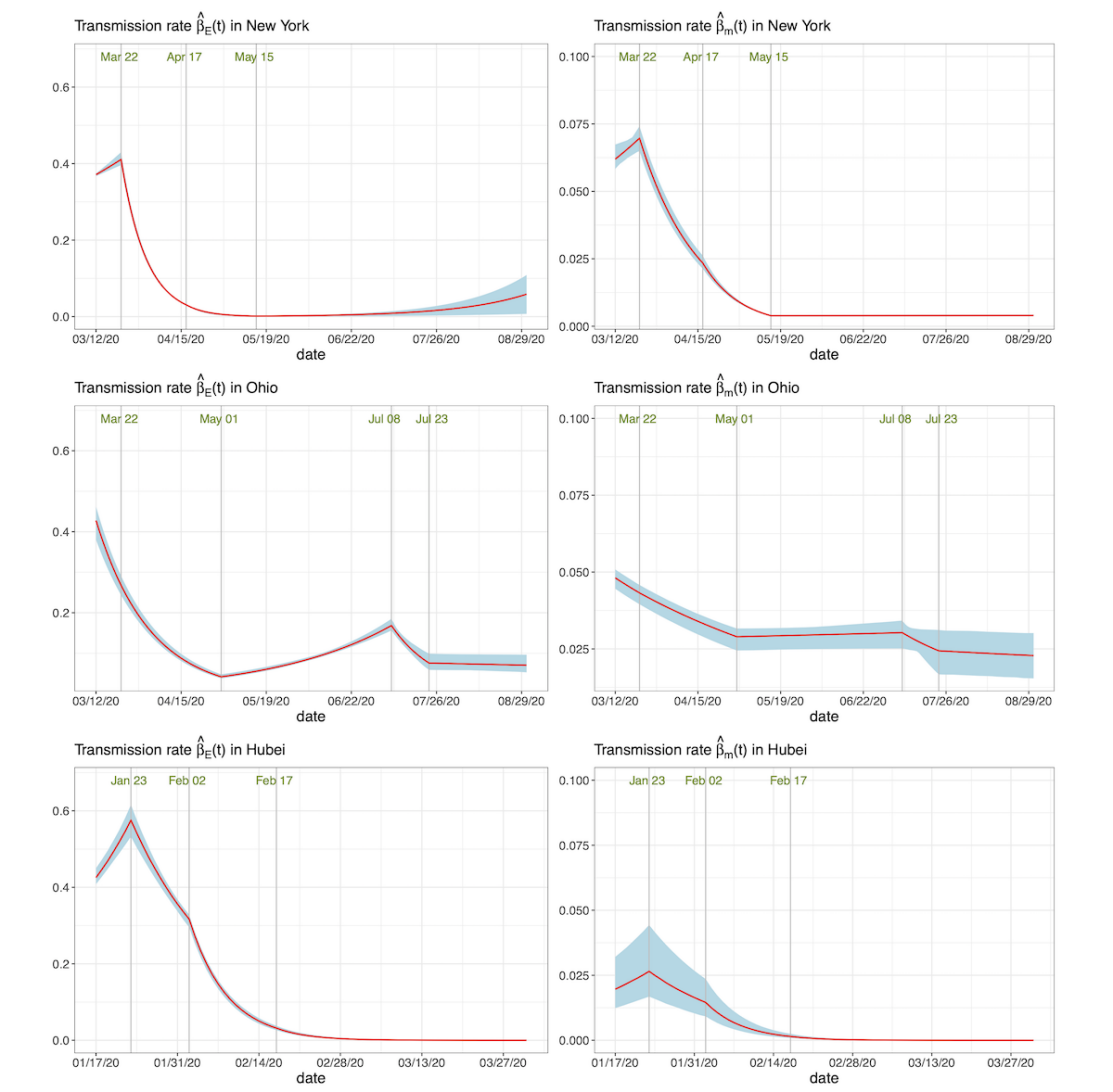
The estimated time-varying transmission rates 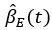 and 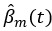, in New York State, Ohio State, and Hubei Province. The solid lines denote the posterior mean curves, and the grey areas denote the 95% credible intervals.

The estimated transmission rate functions 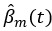 and 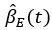 for the three areas are displayed in Figure 3. The estimates of decay parameters with their 95% CIs and the mean differences between different periods are presented in Table S1 in Multimedia Appendix 1.

In New York, the PAUSE Executive Order appeared effective. The transmission rates declined quickly after the order was executed. With the effects of the first and second orders, the rates continued decreasing after April 17, 2020. In Ohio, we noticed that the rates declined before the “stay at home” order because of the early interventions by the government. The transmission rates in Ohio were lower than those in New York before April.

However, the rates in Ohio started to increases after May 1, 2020, when Ohio entered the first phase of its reopening program. Ohio had a second wave of COVID-19 but New York did not in July. It might be because more strict reopening policies were implemented in New York after May. Ohio’s transmission rates decreased again after the order of mandatory use of masks and face coverings was implemented.

In Hubei, the transmission rate of the virus was growing rapidly in the first period because of the large floating population during the Spring Festival of China. In the second period, due to the city lockdown policy implemented, the transmission of the virus had been adequately controlled. In the third period, the transmission rates were further reduced because temporary hospitals effectively treated patients with mild symptoms and isolated the infection sources.

In all areas, the transmission rate of exposed individuals was higher than that of mild patients. The transmission rates of mild patients *β_m_* in New York and Ohio were higher than in Hubei, which may be due to the policy of centralized treatment of mild patients in temporary hospitals in Hubei. This policy seemed to effectively reduce the contact between mild illness patients and susceptible people, thus reducing the transmission rate of mild patients. The public health interventions of social distancing and home confinement played a significant role in preventing and controlling the disease in all areas. The decay rates of transmission were significantly changed (in a Bayesian sense) before and after implementing the interventions (Table S1 in Multimedia Appendix 1).

**Figure 4:**
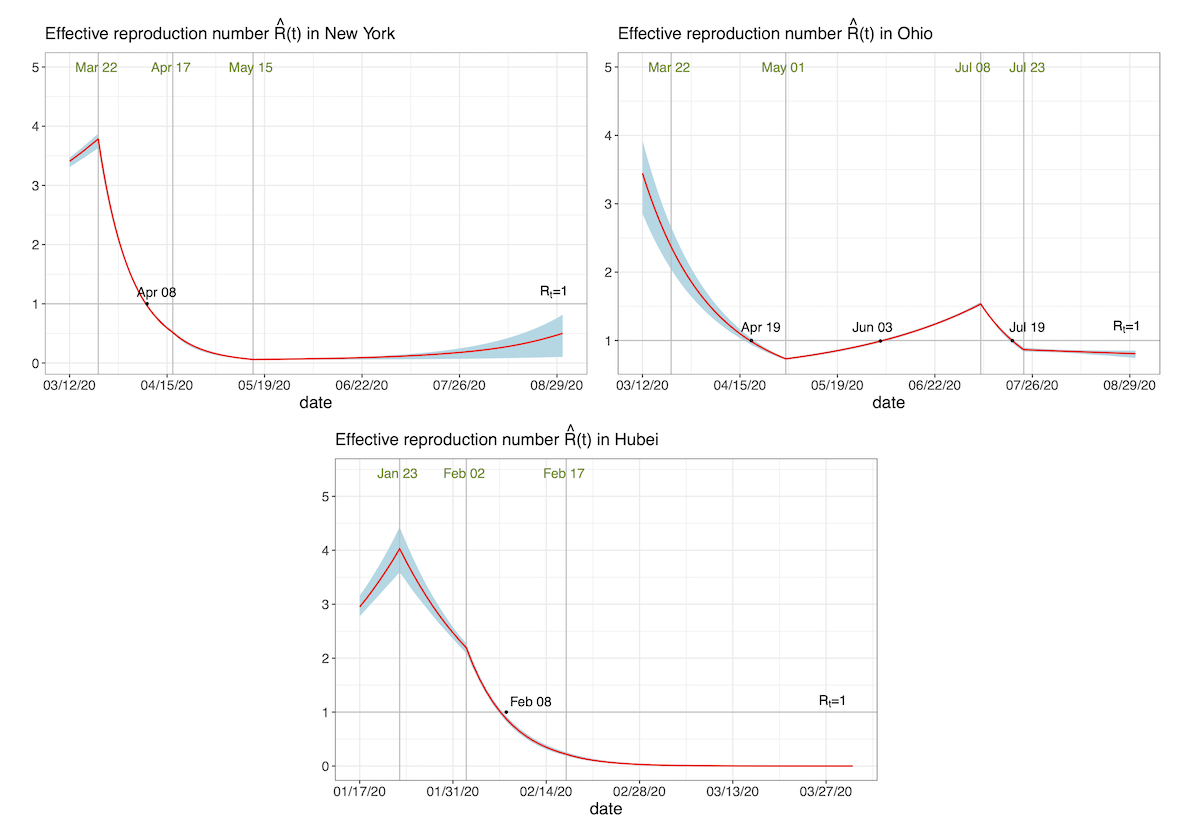
The estimated effective reproduction number 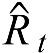 in the three areas. The solid lines denote the posterior mean curves, and the grey areas denote the 95% credible intervals.

The estimated effective reproduction numbers 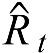 in the three areas are displayed in Figure 4. The solid lines indicate the posterior mean curves, and the grey areas denote the 95% CIs. After the polices of social distancing, no gathering activities, and closure of business activities, 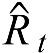 gradually fell below 1.0 for all areas. However, in Ohio, 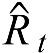 gradually increased after entering the first phase of its reopening program, and exceeded 1.0 on June 3, 2020. It started to decrease after people were mandatorily required to use masks in public.

### Estimated Parameters for COVID-19 Clinical Progression and Disease Severity

Table 1 presents the estimated parameters and their 95% CIs for COVID-19 clinical progression and disease severity. The mean incubation periods were close in the three areas (New York: 6.99 days; Ohio: 6.20 days; Hubei: 6.55 days). However, the time from symptoms to hospital admission in New York and Ohio was longer than that in Hubei (16.84 days and 16.72 days vs 10.20 days, respectively). The time from hospital admission to critical care was shorter in New York and Ohio than that in Hubei (5.43 days and 5.32 days vs 8.49 days, respectively). The time from hospital admission to death was shorter in New York and Ohio than that in Hubei (10.33 days and 11.25 days vs 13.40 days, respectively). The proportion of infected subjects progressing to the severe stage in New York, Ohio, and Hubei were 23.56%, 12.63%, and 30.92%, respectively. The proportions progressing to the critical stage were 14.91%, 6.41%, and 13.49%, respectively. The proportions progressing to the death stage were 8.00%, 5.17%, and 4.48%, respectively. These differences may be because of the different hospitalization and ICU admission criteria, and a mixed patient population.

**Table 1:** Posterior mean and 95% CIs for quantities of COVID-19 clinical progression and disease severity.

Quantities	New York State, posterior mean (95% CI^a^)	Ohio State, posterior mean (95% CI)	Hubei Province, posterior mean (95% CI)
Incubation period (days)	6.99 (6.84-7.17)	6.20 (5.55-6.91)	6.55 (6.28-7.00)
Time from symptoms to hospital admission (days)	16.84 (16.68-16.91)	16.72 (16.28-17.11)	10.20 (9.57-10.81)
Time from hospital admission to critical care (days)	5.43 (5.06-5.80)	5.32 (5.03-5.59)	8.49 (7.77-9.31)
Time from hospital admission to death (days)	10.33 (9.90-10.67)	11.25 (10.52-12.00)	13.40 (12.33-14.52)
Proportion of infected patients progressing to severe stage (%)	23.56 (23.05-24.29)	12.63 (12.02-13.24)	30.92 (29.01-32.88)
Proportion of infected patients progressing to critical stage (%)	14.91 (14.11-15.88)	6.41 (5.76-7.18)	13.49 (12.01-15.05)
Proportion of infected patients progressing to death stage (%)	8.00 (7.88-8.12)	5.17 (4.97-5.37)	4.48 (4.10-4.87)

^a^CI: credible interval.

### Prediction of Hospital Use

Since the COVID-19 pandemic in the United States was still ongoing, we applied our model to predict hospital use since September 1, 2020. Table S4 in Multimedia Appendix 1 presents the predicted versus observed numbers of patients that were newly hospitalized with COVID-19 in New York and Ohio. The mean absolute percentage error (the common measure of prediction accuracy for forecasting) was reasonably low, with 15.15% (SE 3.57%) in New York and 2.07% (SE 0.42%) in Ohio.

## Discussion

New York, Ohio, and Hubei were three representative areas hit by COVID-19 in the United States and China. Evaluating the effectiveness of their public health interventions on reducing disease burden and estimating hospital use through the available historical epidemiological data is vital since adequate hospital capacity is critical to saving lives during this ongoing pandemic. Our findings highlight that early intervention is crucial. Ohio has lower transmission rates than New York in the early stage of the pandemic because Ohio declared a state of emergency on March 9, 2020. We also found that wearing masks is critical in controlling disease transmission. Ohio had a second peak of the outbreak but New York did not after entering the phase of reopening. In comparing their policies, Ohio implemented the order of the mandatory use of masks in July, while New York implemented it in April.

We noticed that the centralized quarantine, in addition to social distancing and wearing masks, could play an important and indispensable role in preventing and controlling the disease. In Hubei, the decay parameters in 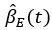 and 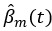 significantly increased after the centralized quarantine was implemented, which indicated the disease transmission received further controls. During Hubei’s lockdown, more than sixteen public venues such as exhibition centers and gymnasiums were converted into temporary hospitals to treat patients with mild symptoms and isolate the source of infections amid strained medical resources. Before these hospitals, many confirmed cases were quarantined at home and stayed in their communities, which could easily lead to clustered infections in families and communities. In New York, there was no centralized quarantine intervention and the mild patients were required to quarantine at home. A recent survey of nearly 1300 patients with COVID-19 admitted to hospitals in New York in May 2020 showed that 83% of new patients were at home. The temporary hospitals that treat mild patients during the COVID-19 epidemic could be beneficial in effectively reducing the contact of mild illness patients and susceptible people, and further reducing the transmission rate.

Consistent with the results from the recent studies in Wuhan City, China [14,15,25,26], our results showed that public health interventions dramatically decreased the case incidence and reduced the demand for hospital and ICU beds in these areas. In Figures S3 and S4 in Multimedia Appendix 1, we demonstrate a few simulation scenarios when interventions were not implemented. We found that COVID-19 would overwhelm the hospital bed capacity limits if the interventions were delayed or not implemented. One caveat in this conclusion is that our analyses looked at bed capacity and use at a state level. Health care use, however, is local: unless bed use is coordinated across the multiple hospital systems within a given state, patients may overwhelmingly “flock” to a handful of specialized hospitals and overwhelm them. In such situations, even if there is adequate capacity across the state, individual hospital systems may be overwhelmed. This calls attention to the importance of public health coordination of resources and strategies.

At present, the immunogenicity, efficacy, safety, production capacity, and availability of COVID-19 vaccines are not yet clear. It is crucial to maintain the transmission at a low level until a safe and effective COVID-19 vaccine is developed and widely used to establish a population immune barrier. The recent meta-analysis by Chu et al [27] confirmed evidence of moderate certainty that current policies of at least 1 m physical distancing were probably associated with a large reduction in infection and that distances of 2 m might be more effective. The public health interventions, including social distancing and wearing a face covering, should be continued to avoid the next peak of the outbreak.

The advantage of our proposed statistical methods is that all dynamic parameters can be estimated from observed data using MCMC algorithms under the Bayesian modeling framework. It is unlike many other methods that simulated disease spread with prespecified parameters. We hope our analysis provides data-driven evidence to potentially improve on whether, when, and how to adapt public health interventions in the future. As an increasing number of cases are still being identified in the United States, we believe our proposed model could help project hospital use during the COVID-19 outbreaks.

## Appendix

Multimedia Appendix 1 Supplementary material.

## Abbreviations

CI: credible interval
ICU: intensive care unit
MCMC: Markov chain Monte Carlo
PAUSE: Policies Assure Uniform Safety for Everyone
SEIR: susceptible-exposed-infectious-removed
WHO: World Health Organization
