# Supplementary material for: Public Health Interventions’ Effect on Hospital Use in Patients With COVID-19: Comparative Study
Source: JMIR Public Health Surveill. 2020 Dec 23;6(4):e25174. doi: 10.2196/25174 (PMC7759508; doi:10.2196/25174)

***Supplement for “Effects of Public Health Interventions on Hospital Utilization in Patients with COVID-19: a Comparative Study”***

# Dynamic SEIR model: the technical details

From the main paper, our model is expressed as a system of ordinary differential equations:

$${\frac{dS}{dt}=\delta R-\frac{\left( \beta_{m}I_{m}+\beta_{E}E \right)S}{N} \atop\begin{aligned} \frac{dE}{dt}=\frac{\left( \beta_{m}I_{m}+\beta_{E}E \right)S}{N}-\left( \alpha_{m}+\alpha_{s} \right)E \\ \frac{dI_{m}}{dt}=\alpha_{m}E-\left( \gamma_{m}+p_{s} \right)I_{m} \\ \frac{dI_{s}}{dt}=\alpha_{s}E+p_{s}I_{m}-\left( \gamma_{s}+p_{c}+q_{s} \right)I_{s} \\ \frac{dI_{c}}{dt}=p_{c}I_{s}-\left( \gamma_{c}+q_{c} \right)I_{c} \\ \frac{dR}{dt}=\gamma_{m}I_{m}+\gamma_{s}I_{s}+\gamma_{c}I_{c}-\delta R \\ \frac{dD}{dt}={q_{s}I}_{s}+{q_{c}I}_{c} \end{aligned} (1)}$$

with the size of population $N$:

$$N=S\left( t \right)+E\left( t \right)+I_{m}\left( t \right)+I_{s}\left( t \right)+I_{c}\left( t \right)+R\left( t \right)+D\left( t \right).$$

We make the following transformation:

$$s=\frac{S}{N}, e=\frac{E}{N}, i_{m}=\frac{I_{m}}{N}, i_{s}=\frac{I_{s}}{N}, i_{c}=\frac{I_{c}}{N}, r=\frac{R}{N}, g=\frac{D}{N},$$

where $s$, $e$, $i_{m}$, $i_{s}$, $i_{c}$, $r$ and $g$ denote the fractions of the number of individuals in classes $S$, $E$ $I_{m}$, $I_{s}$, $I_{c}$, $R$ and $D$ with population $N$. So, the transformed system is given by

$${\frac{ds}{dt}=\delta r-\left( \beta_{m}i_{m}+\beta_{E}e \right)s \atop\begin{aligned} \frac{de}{dt}=\left( \beta_{m}i_{m}+\beta_{E}e \right)s-\left( \alpha_{m}+\alpha_{s} \right)e \\ \frac{di_{m}}{dt}=\alpha_{m}e-\left( \gamma_{m}+p_{s} \right)i_{m} \\ \frac{di_{s}}{dt}=\alpha_{s}e+p_{s}i_{m}-\left( \gamma_{s}+p_{c}+q_{s} \right)i_{s} (2) \\ \frac{di_{c}}{dt}=p_{c}i_{s}-\left( \gamma_{c}+q_{c} \right)i_{c} \\ \frac{dr}{dt}=\gamma_{m}i_{m}+\gamma_{s}i_{s}+\gamma_{c}i_{c}-\delta r \\ \frac{dg}{dt}=q_{s}i_{s}+q_{c}i_{c} \end{aligned}}$$

## Modeling the effects of public health interventions

In order to reflect the governmental public health policy effects in the different time periods, we model the transmission rates using piecewise exponential functions. Specifically, for New York State and Hubei Province,

$$\beta_{j}(t)=\left\{ \begin{aligned} \beta_{j}^{*}e^{-k_{j1}t}, for t\in[T_{0},T_{1}] \\ \beta_{j}^{*}e^{-\left( k_{j1}-k_{j2} \right)T_{1}}\cdot e^{-k_{j2}t}, for t\in\left( T_{1},T_{2} \right] \\ \beta_{j}^{*}e^{-\left( k_{j1}-k_{j2} \right)T_{1}-\left( k_{j2}-k_{j3} \right)T_{2}}\cdot e^{-k_{j3}t}, for t\in\left( T_{2},T_{3} \right] \\ \beta_{j}^{*}e^{-\left( k_{j1}-k_{j2} \right)T_{1}-\left( k_{j2}-k_{j3} \right)T_{2}-\left( k_{j3}-k_{j4} \right)T_{3}}\cdot e^{-k_{j4}t}, for t\in\left( T_{3},T_{4} \right] \end{aligned} \right.$$

where $\beta_{j}(t)$ denotes either $\beta_{m}(t)$ or $\beta_{E}(t)$, $\beta_{j}^{*}$ represents the baseline value of $\beta_{j}(t)$, and $k_{j1}$, $k_{j2}$, $k_{j3}$ and $k_{j4}$ are the decay parameters to be estimated in the two areas. For Ohio,

$$\beta_{j}(t)=\left\{ \begin{aligned} \beta_{j}^{*}e^{-k_{j1}t}, for t\in\left[ T_{0},T_{1} \right] \\ \beta_{j}^{*}e^{-\left( k_{j1}-k_{j2} \right)T_{1}}\cdot e^{-k_{j2}t}, for t\in\left( T_{1},T_{2} \right] \\ \beta_{j}^{*}e^{-\left( k_{j1}-k_{j2} \right)T_{1}-\left( k_{j2}-k_{j3} \right)T_{2}}\cdot e^{-k_{j3}t}, for t\in\left( T_{2},T_{3} \right] \\ \beta_{j}^{*}e^{-\left( k_{j1}-k_{j2} \right)T_{1}-\left( k_{j2}-k_{j3} \right)T_{2}-\left( k_{j3}-k_{j4} \right)T_{3}}\cdot e^{-k_{j4}t}, for t\in\left( T_{3},T_{4} \right] \\ \beta_{j}^{*}e^{-\left( k_{j1}-k_{j2} \right)T_{1}-\left( k_{j2}-k_{j3} \right)T_{2}-\left( k_{j3}-k_{j4} \right)T_{3}-\left( k_{j4}-k_{j5} \right)T_{4}}\cdot e^{-k_{j5}t}, for t\in\left( T_{4},T_{5} \right] \end{aligned} \right.$$

where $\beta_{j}^{*}$, $k_{j1}$, $k_{j2}$, $k_{j3}$, $k_{j4}$ and $k_{j5}$ are the parameters to be estimated.

We model the cure rate $\gamma_{m}(t)$ with a logistic function in order to reflect the continuous improvement of reactive medical resources in both areas:

$$\gamma_{m}\left( t \right)=\frac{\gamma_{m}^{*}\gamma_{0}^{*}e^{r(t-t^{*})}}{\gamma_{m}^{*}+\gamma_{0}^{*}\left[ e^{r(t-t^{*})}-1 \right]}$$

where $\gamma_{0}^{*}$ is the initial value at $t^{*}$, $\gamma_{m}^{*}$ is the maximum value of $\gamma_{m}$, and $r$ measures the growth rate of the curve $\gamma_{m}\left( t \right)$. Here we set $t^{*}=14$, the average time for the mild patients to recover, and $\gamma_{0}^{*}$, $\gamma_{m}^{*}$, and $r$ are the parameters to be estimated.

## Estimating the parameters for the model in Hubei Province

We begin with an epidemiological model in the framework of state-space SEIR models with no consideration of quarantine protocols as shown in Figure 1. In Hubei Province, we consider five time series of proportions of mild infected ($I_{m}$), severe infected ($I_{s}$), critical infected ($I_{c}$), removed cases ($R$) and dead cases ($D$), denoted by $Y=(Y_{t}^{I_{m}}, Y_{t}^{I_{s}},Y_{t}^{I_{c}}, Y_{t}^{R},Y_{t}^{D})$ at time $t$, respectively. We assume that the 5-dimensional time series of $Y$ follows a state-space model with the beta distributions at time $t$:

$${Y_{t}^{I_{m}}|\boldsymbol{\theta}_{t},\boldsymbol{\tau} \sim\mathrm{Beta}\left( \lambda^{I_{m}}\theta_{t}^{I_{m}},\lambda^{I_{m}}\left( 1-\theta_{t}^{I_{m}} \right) \right) \atop\begin{aligned} Y_{t}^{I_{s}}|\boldsymbol{\theta}_{t},\boldsymbol{\tau} \sim\mathrm{Beta}\left( \lambda^{I_{s}}\theta_{t}^{I_{s}},\lambda^{I_{s}}\left( 1-\theta_{t}^{I_{s}} \right) \right) \\ Y_{t}^{I_{c}}|\boldsymbol{\theta}_{t},\boldsymbol{\tau} \sim\mathrm{Beta}\left( \lambda^{I_{c}}\theta_{t}^{I_{c}},\lambda^{I_{c}}\left( 1-\theta_{t}^{I_{c}} \right) \right) \\ Y_{t}^{R}|\boldsymbol{\theta}_{t},\boldsymbol{\tau} \sim\mathrm{Beta}\left( \lambda^{R}\theta_{t}^{R},\lambda^{R}\left( 1-\theta_{t}^{R} \right) \right) \\ Y_{t}^{D}|\boldsymbol{\theta}_{t},\boldsymbol{\tau} \sim\mathrm{Beta}\left( \lambda^{D}\theta_{t}^{D},\lambda^{D}\left( 1-\theta_{t}^{D} \right) \right) \end{aligned}}$$

where $\theta_{t}^{I_{m}}$, $\theta_{t}^{I_{s}}$, $\theta_{t}^{I_{c}}$, $\theta_{t}^{R}$ and $\theta_{t}^{D}$ are the respective prevalence at each corresponding stage at time $t$, and $\lambda^{I_{m}}$, $\lambda^{I_{s}}$, $\lambda^{I_{c}}$, $\lambda^{R}$ and $\lambda^{D}$ are the parameters controlling the respective variances of the observed proportions.

Assume that these observed time series are emitted from the underlying latent dynamics of COVID-19 infection characterized by the latent Markov process $\boldsymbol{\theta}_{t}$. Obviously, the expected proportions are equal to the prevalence at each corresponding stage at time $t$,

$${E\left( Y_{t}^{I_{m}}|\boldsymbol{\theta}_{t} \right)=\theta_{t}^{I_{m}} \atop\begin{aligned} E\left( Y_{t}^{I_{s}}|\boldsymbol{\theta}_{t} \right)=\theta_{t}^{I_{s}} \\ E\left( Y_{t}^{I_{c}}|\boldsymbol{\theta}_{t} \right)=\theta_{t}^{I_{c}} \\ E\left( Y_{t}^{R}|\boldsymbol{\theta}_{t} \right)=\theta_{t}^{R} \\ E\left( Y_{t}^{D}|\boldsymbol{\theta}_{t} \right)=\theta_{t}^{D} \end{aligned}}$$

Moreover, the latent population prevalence $\boldsymbol{\theta}_{t}=\left( \theta_{t}^{S},\theta_{t}^{E},\theta_{t}^{I_{m}},\theta_{t}^{I_{s}},\theta_{t}^{I_{c}},\theta_{t}^{R},\theta_{t}^{D} \right)$ is a 7-dimensional Markov process, in which $\theta_{t}^{*}$ is the probability of a person belonging to one stage of $\left( S,E,I_{m},I_{m},I_{m},R,D \right)$ at time $t$. Here, $\theta_{t}^{S}+\theta_{t}^{E}+\theta_{t}^{I_{m}}+\theta_{t}^{I_{s}}+\theta_{t}^{I_{c}}+\theta_{t}^{R}+\theta_{t}^{D}=1$. We assume that this 7-dimensional prevalence process $\boldsymbol{\theta}_{t}$ is governed by the following DSEIR model:

$${\frac{d\theta_{t}^{S}}{dt}=\delta\theta_{t}^{R}-\left( \beta_{m}(t)\theta_{t}^{I_{m}}+\beta_{E}(t)\theta_{t}^{E} \right)\theta_{t}^{S} \atop\begin{aligned} \frac{d\theta_{t}^{E}}{dt}=\left( \beta_{m}(t)\theta_{t}^{I_{m}}+\beta_{E}(t)\theta_{t}^{E} \right)\theta_{t}^{S}-\left( \alpha_{m}+\alpha_{s} \right)\theta_{t}^{E} \\ \frac{d\theta_{t}^{I_{m}}}{dt}=\alpha_{m}\theta_{t}^{E}-\left( \gamma_{m}(t)+p_{s} \right)\theta_{t}^{I_{m}} \\ \frac{d\theta_{t}^{I_{s}}}{dt}=\alpha_{s}\theta_{t}^{E}+p_{s}\theta_{t}^{I_{m}}-\left( \gamma_{s}+p_{c}+q_{s} \right)\theta_{t}^{I_{s}} \\ \frac{d\theta_{t}^{I_{c}}}{dt}=p_{c}\theta_{t}^{I_{s}}-\left( \gamma_{c}+q_{c} \right)\theta_{t}^{I_{c}} \\ \frac{d\theta_{t}^{R}}{dt}=\gamma_{m}(t)\theta_{t}^{I_{m}}+\gamma_{s}\theta_{t}^{I_{s}}+\gamma_{c}\theta_{t}^{I_{c}}-\delta\theta_{t}^{R} \\ \frac{d\theta_{t}^{D}}{dt}=q_{s}\theta_{t}^{I_{s}}+q_{c}\theta_{t}^{I_{c}} \end{aligned}}$$

Denote the vector $\boldsymbol{\tau}_{\text{HB}}=\left( \beta_{m}^{*},\beta_{E}^{*},\alpha_{m},\alpha_{s},\gamma_{m}^{*},\gamma_{0}^{*},\gamma_{s},\gamma_{c},p_{s},p_{c},q_{s},q_{c},\delta,k_{1\sim4},r,\lambda^{I_{m}},\lambda^{I_{s}},\lambda^{I_{c}},\lambda^{R},\lambda^{D} \right)$ as the set of model parameters to be estimated. Then, we use the Markov chain Monte Carlo method (MCMC) to estimate the posterior distribution of $\boldsymbol{\tau}_{\text{HB}}$ and compute Bayesian quantities of interest. We use the data from January 17 to March 31 to train the model and estimate the coefficients. However, the observed data is not complete all over time. The data $(Y_{t}^{I_{m}}, Y_{t}^{I_{s}},Y_{t}^{I_{c}})$ from January 17 to January 25 are not observed. Therefore, in actual calculation, the dynamic model from January 17 to January 25 is trained only with data set $( Y_{t}^{R},Y_{t}^{D})$, and the model from January 26 to March 31 is trained with complete data set $(Y_{t}^{I_{m}}, Y_{t}^{I_{s}},Y_{t}^{I_{c}}, Y_{t}^{R},Y_{t}^{D})$.

## Estimating the parameters for the model in Ohio State

Similar to the model of Hubei province established in section 1.2, in New York State, we consider five time series of proportions of mild infected ($I_{m}$), severe infected ($I_{s}$), critical infected ($I_{c}$), removed cases ($R$) and dead cases ($D$), denoted by $Y=(Y_{t}^{I_{m}}, Y_{t}^{I_{s}},Y_{t}^{I_{c}}, Y_{t}^{R},Y_{t}^{D})$ at time $t$, respectively, and assume that the 5-dimensional time series of $Y$ follows a state-space model with the beta distributions at time $t$:

$${Y_{t}^{I_{m}}|\boldsymbol{\theta}_{t},\boldsymbol{\tau} \sim\mathrm{Beta}\left( \lambda^{I_{m}}\theta_{t}^{I_{m}},\lambda^{I_{m}}\left( 1-\theta_{t}^{I_{m}} \right) \right) \atop\begin{aligned} Y_{t}^{I_{s}}|\boldsymbol{\theta}_{t},\boldsymbol{\tau} \sim\mathrm{Beta}\left( \lambda^{I_{s}}\theta_{t}^{I_{s}},\lambda^{I_{s}}\left( 1-\theta_{t}^{I_{s}} \right) \right) \\ Y_{t}^{I_{c}}|\boldsymbol{\theta}_{t},\boldsymbol{\tau} \sim\mathrm{Beta}\left( \lambda^{I_{c}}\theta_{t}^{I_{c}},\lambda^{I_{c}}\left( 1-\theta_{t}^{I_{c}} \right) \right) \\ Y_{t}^{R}|\boldsymbol{\theta}_{t},\boldsymbol{\tau} \sim\mathrm{Beta}\left( \lambda^{R}\theta_{t}^{R},\lambda^{R}\left( 1-\theta_{t}^{R} \right) \right) \\ Y_{t}^{D}|\boldsymbol{\theta}_{t},\boldsymbol{\tau} \sim\mathrm{Beta}\left( \lambda^{D}\theta_{t}^{D},\lambda^{D}\left( 1-\theta_{t}^{D} \right) \right) \end{aligned}}$$

Then the vector $\boldsymbol{\tau}_{\text{OH}}\mathbf{=}\left( \beta_{m}^{*},\beta_{E}^{*},\alpha_{m},\alpha_{s},\gamma_{m}^{*},\gamma_{0}^{*},\gamma_{s},\gamma_{c},p_{s},p_{c},q_{s},q_{c},\delta,k_{1\sim5},r,\lambda^{I_{a}},\lambda^{I_{m}},\lambda^{I_{h}},\lambda^{R},\lambda^{D} \right)$ is the set of parameters to be estimated. Similarly, we use the MCMC to estimate the posterior distribution of $\boldsymbol{\tau}_{\text{OH}}$ and compute Bayesian quantities of interest. Since the data $(Y_{t}^{I_{m}}, Y_{t}^{I_{s}},Y_{t}^{I_{c}})$ from March 12 to May 1 are not observed, then the dynamic model from March 12 to May 1 is trained only with data set $( Y_{t}^{R},Y_{t}^{D})$, and the model from May 2 to August 31 is trained with complete data set $(Y_{t}^{I_{m}}, Y_{t}^{I_{s}},Y_{t}^{I_{c}}, Y_{t}^{R},Y_{t}^{D})$.

## Estimating the parameters for the model in New York State

In New York State, we have made some changes, comparing with Hubei and Ohio, since we only observed five time series $Y_{0}$ of proportions of cumulative confirmed cases ($I_{a}=I_{m}+I_{s}+I_{c}+R+D$), current confirmed cases ($I_{b}=I_{m}+I_{s}+I_{c}$), hospitalized infected ($I_{h}=I_{s}+I_{c}$), removed cases ($R$) and dead cases ($D$), denote by $Y_{0}=\left( Y_{t}^{I_{a}},Y_{t}^{I_{b}},Y_{t}^{I_{h}},Y_{t}^{R},Y_{t}^{D} \right)$. We approximate the time series of proportions of mild infected as $Y_{t}^{I_{m}}=Y_{t}^{I_{b}}-Y_{t}^{I_{h}}$, and assume that the 5-dimensional time series of $Y=\left( Y_{t}^{I_{a}},Y_{t}^{I_{m}},Y_{t}^{I_{h}},Y_{t}^{R},Y_{t}^{D} \right)$ follows a state-space model with the beta distributions at time $t$:

$${\begin{aligned} Y_{t}^{I_{a}}|\boldsymbol{\theta}_{t},\boldsymbol{\tau} \sim\mathrm{Beta}\left( \lambda^{I_{a}}\theta_{t}^{I_{a}},\lambda^{I_{a}}\left( 1-\theta_{t}^{I_{a}} \right) \right) \\ Y_{t}^{I_{m}}|\boldsymbol{\theta}_{t},\boldsymbol{\tau} \sim\mathrm{Beta}\left( \lambda^{I_{m}}\theta_{t}^{I_{m}},\lambda^{I_{m}}\left( 1-\theta_{t}^{I_{m}} \right) \right) \end{aligned} \atop\begin{aligned} Y_{t}^{I_{h}}|\boldsymbol{\theta}_{t},\boldsymbol{\tau} \sim\mathrm{Beta}\left( \lambda^{I_{h}}\theta_{t}^{I_{h}},\lambda^{I_{h}}\left( 1-\theta_{t}^{I_{h}} \right) \right) \\ Y_{t}^{R}|\boldsymbol{\theta}_{t},\boldsymbol{\tau} \sim\mathrm{Beta}\left( \lambda^{R}\theta_{t}^{R},\lambda^{R}\left( 1-\theta_{t}^{R} \right) \right) \\ Y_{t}^{D}|\boldsymbol{\theta}_{t},\boldsymbol{\tau} \sim\mathrm{Beta}\left( \lambda^{D}\theta_{t}^{D},\lambda^{D}\left( 1-\theta_{t}^{D} \right) \right) \end{aligned}}$$

Similar to Section 1.2, the prevalence $\boldsymbol{\theta}_{t}=\left( \theta_{t}^{S},\theta_{t}^{E},\theta_{t}^{I_{m}},\theta_{t}^{I_{s}},\theta_{t}^{I_{c}},\theta_{t}^{R},\theta_{t}^{D} \right)$ is a 7-dimensional Markov process, in which $\theta_{t}^{*}$ is the probability of a person belonging to one stage of $\left( S,E,I_{m},I_{s},I_{c},R,D \right)$ at time $t$. In addition, suppose that $\theta_{t}^{I_{a}}=\theta_{t}^{I_{m}}+\theta_{t}^{I_{s}}+\theta_{t}^{I_{c}}+\theta_{t}^{R}+\theta_{t}^{D}$ is the prevalence for total confirmed population and $\theta_{t}^{I_{h}}=\theta_{t}^{I_{s}}+\theta_{t}^{I_{c}}$ is for hospitalized population. The 7-dimensional prevalence process $\boldsymbol{\theta}_{t}$ is governed by the following DSEIR model which is the same as that in Section 1.2.

The vector $\boldsymbol{\tau}_{\mathrm{NY}}\mathbf{=}\left( \beta_{m}^{*},\beta_{E}^{*},\alpha_{m},\alpha_{s},\gamma_{m}^{*},\gamma_{0}^{*},\gamma_{s},\gamma_{c},p_{s},p_{c},q_{s},q_{c},\delta,k_{1\sim4},r,\lambda^{I_{a}},\lambda^{I_{m}},\lambda^{I_{h}},\lambda^{R},\lambda^{D} \right)$ is the set of parameters to be estimated. Similarly, we use the MCMC to estimate the posterior distribution of $\boldsymbol{\tau}_{\mathrm{NY}}$ and compute Bayesian quantities of interest. The data $(Y_{t}^{I_{m}}, Y_{t}^{I_{h}})$ from March 12 to March 20 are not observed. In actual calculation, the dynamic model from March 12 to March 20 is trained only with data set $( Y_{t}^{a},Y_{t}^{R},Y_{t}^{D})$, and the model from January 26 to March 31 is trained with complete data set $(Y_{t}^{I_{a}}, Y_{t}^{I_{m}},Y_{t}^{I_{h}}, Y_{t}^{R},Y_{t}^{D})$.

1. **Model fitting for the epidemic trend of COVID-19 in New York State, Ohio and Hubei Province.**

**Figure S1: Curve fitting for the epidemic trend of COVID-19 in New York State and Hubei Province: the dots denote the observed data, and the curves denote the estimated curved by the models.**

**Table S1: Posterior means with 95% credible intervals for the parameters in the dynamic SEIR models.**

| Parameter | New York | Ohio | Hubei Province |
| --- | --- | --- | --- |
| $\gamma_{m}^{*}$ | 0.0593(0.0590, 0.0598) | 0.0537(0.0525,0.0536) | 0.0828(0.0770, 0.0904) |
| $\gamma_{s}$ | 0.0487(0.0481, 0.0490) | 0.0541(0.0502,0.0631) | 0.0602(0.0516, 0.0697) |
| $\gamma_{c}$ | 0.1277(0.1206, 0.1345) | 0.1010(0.0929,0.1092) | 0.1637(0.1461, 0.1825) |
| $p_{s}$ | 0.0001(0.0001, 0.0002) | 0.0061(0.0055,0.0067) | 0.0153(0.0120, 0.0185) |
| $p_{c}$ | 0.1167(0.1075, 0.1297) | 0.0955(0.0842,0.1076) | 0.0515(0.0452, 0.0580) |
| $\alpha_{m}$ | 0.1094(0.1060, 0.1123) | 0.1576(0.1403,0.1753) | 0.1251(0.1167, 0.1298) |
| $\alpha_{s}$ | 0.0335(0.0330, 0.0346) | 0.0043(0.0030,0.0056) | 0.0277(0.0239, 0.0317) |
| $q_{s}$ | 0.0189(0.0138, 0.0249) | 0.0386(0.0292,0.0474) | 0.0063(0.0045, 0.0070) |
| $q_{c}$ | 0.0767(0.0653, 0.0873) | 0.0680(0.0525,0.0857) | 0.0434(0.0352, 0.0541) |
| $\delta$ | 5.3E-7(2.2E-8, 9.8E-7) | 5.3E-7(3.9E-8,9.8E-7) | 5.4E-8(4.8E-10, 4.8E-6) |

# Parameters in time-varying transmission rates ${\hat{\boldsymbol{\beta}}}_{\boldsymbol{E}}\boldsymbol{(t)}$ and ${\hat{\boldsymbol{\beta}}}_{\boldsymbol{m}}\boldsymbol{(t)}$.

**Table S2: Estimates of the decay parameters and their differences in time-varying transmission rates** ${\hat{\boldsymbol{\beta}}}_{\boldsymbol{E}}\boldsymbol{(t)}$ **and** ${\hat{\boldsymbol{\beta}}}_{\boldsymbol{m}}\boldsymbol{(t)}$**.**

|  | Time period | New York | Ohio | Hubei Province |
| --- | --- | --- | --- | --- |
| $\beta_{m}(t)$ |  |  |  |  |
| $k_{m1}$ | $T_{0}\sim T_{1}$ | -0.0119(-0.0203, -0.0010) | 0.0107(0.0080, 0.0133) | -0.0507(-0.0595, -0.0402) |
| $k_{m2}$ | $T_{1}\sim T_{2}$ | 0.0420(0.0384, 0.0449) | 0.0101(0.0077, 0.0121) | 0.0603(0.0513, 0.0708) |
| $k_{m3}$ | $T_{2}\sim T_{3}$ | 0.0639(0.0597, 0.0637) | -0.0007(-0.0021, -0.0001) | 0.1531(0.1408, 0.1642) |
| $k_{m4}$ | $T_{3}\sim T_{4}$ | -0.0002(-0.0007, -0.0001) | 0.0151(0.0005, 0.0439) | 0.1798(0.1525, 0.2055) |
| $k_{m5}$ | $T_{4}\sim T_{5}$ | -- | 0.0018(0.0000, 0.0062) | -- |
|  |  |  |  |  |
| $\beta_{E}(t)$ |  |  |  |  |
| $k_{E1}$ | $T_{0}\sim T_{1}$ | -0.0101(-0.0139, -0.0072) | 0.0469(0.0447, 0.0483) | -0.0445(-0.0591, -0.0308) |
| $k_{E2}$ | $T_{1}\sim T_{2}$ | 0.0995(0.0987, 0.1000) | 0.0464(0.0444, 0.0475) | 0.1099(0.0923, 0.1304) |
| $k_{E3}$ | $T_{2}\sim T_{3}$ | 0.1173(0.0994, 0.1574) | -0.0205(-0.0227, -0.0180) | 0.1590(0.1060, 0.2135) |
| $k_{E4}$ | $T_{3}\sim T_{4}$ | -0.0339(-0.0379, -0.0256) | 0.0538(0.0342, 0.0745) | 0.1844(0.1199, 0.2483) |
| $k_{E5}$ | $T_{4}\sim T_{5}$ | -- | 0.0020(0.0000, 0.0072) | -- |
| $k_{m2}-k_{m1}$ | | 0.0538(0.0435, 0.0623) | -0.0007(-0.0021, -0.0001) | 0.0687(0.0504, 0.0802) |
| $k_{m3}-k_{m2}$ | | 0.0219(0.0158, 0.0295) | -0.0078(-0.0134, -0.0078) | 0.0963(0.0868, 0.0999) |
| $k_{m4}-k_{m3}$ | | -0.0641(-0.0692, -0.0599) | 0.0158(0.0007, 0.0445) | 0.0272(0.0025, 0.0487) |
| $k_{m5}-k_{m4}$ | | -- | -0.0134(-0.0427, -0.0022) | -- |
|  |  |  |  |  |
| $k_{E2}-k_{E1}$ | | 0.1096(0.1068, 0.1134) | -0.0004(-0.0017, -0.0001) | 0.1253(0.1004, 0.1520) |
| $k_{E3}-k_{E2}$ | | 0.0178(0.0003, 0.0578) | -0.0669(-0.0695, -0.0633) | 0.0509(0.0027, 0.0974) |
| $k_{E4}-k_{E3}$ | | -0.1512(-0.1877, -0.1311) | 0.0743(0.0551, 0.0972) | 0.0249(0.0011, 0.0484) |
| $k_{E5}-k_{E4}$ | | -- | -0.0518(-0.0736, -0.0330) | -- |

1. **Time-varying cure rate for mild patients in New York, Ohio and Hubei Province**

**Figure S2: The estimated time-varying cure rate** ${\hat{\boldsymbol{\gamma}}}_{\boldsymbol{m}}\boldsymbol{(t)}$ **in New York State, Ohio and Hubei Province: the solid lines denote the posterior mean curves, and the grey areas denote the 95% credible intervals.**

# Formulas of the quantities for COVID-19 clinical progression and disease severity.

**Table S3:**

| Quantity | Formula |
| --- | --- |
| Incubation period | $1/\left( \alpha_{m}+\alpha_{s} \right)$ |
| Duration of mild infection | $1/\left( \gamma_{m}+p_{s} \right)$ |
| Duration of severe infection | $1/\left( \gamma_{s}+q_{s}+p_{c} \right)$ |
| Duration of critical infection | $1/\left( \gamma_{c}+q_{c} \right)$ |
| Proportion of infected subjects progressing to severe stage | $\frac{\alpha_{s}}{\alpha_{s}+\alpha_{m}}+\frac{\alpha_{m}}{\alpha_{s}+\alpha_{m}}\cdot\frac{p_{s}}{p_{s}+\gamma_{m}}$ |
| Proportion of infected subjects progressing to critical stage | $\left( \frac{\alpha_{s}}{\alpha_{s}+\alpha_{m}}+\frac{\alpha_{m}}{\alpha_{s}+\alpha_{m}}\cdot\frac{p_{s}}{p_{s}+\gamma_{m}} \right)\frac{p_{c}}{q_{s}+p_{c}{+\gamma}_{s}}$ |
| Proportion of infected subjects progressing to death stage | $\left( \frac{\alpha_{s}}{\alpha_{s}+\alpha_{m}}+\frac{\alpha_{m}}{\alpha_{s}+\alpha_{m}}\cdot\frac{p_{s}}{p_{s}+\gamma_{m}} \right)\left( \frac{q_{s}}{q_{s}+p_{c}{+\gamma}_{s}}+\frac{p_{c}}{q_{s}+p_{c}{+\gamma}_{s}}\cdot\frac{q_{c}}{q_{c}+\gamma_{c}} \right)$ |

# Summary Results for the analysis of the effects of public health interventions.

**Figure S3:** The evaluation of the nonpharmaceutical public health interventions' effectiveness by considering the scenario that no interventions are implemented. The solid lines denote the predicted mean curves under different scenarios, and the grey areas denote the 95% credible intervals. And the solid points represent the observed data and the hollow points represent the predicted values in this paper.

**Figure S4:** The evaluation of the nonpharmaceutical public health interventions' effectiveness for Hubei, by considering that the interventions of city lockdown are implemented but without centralized treatment.

1. **Prediction of hospital utilization in New York State.**

**Table S4: Predicted vs Observed numbers of new hospitalized patients with COVID-19 in New York State and Ohio since September 1, 2020. The MAPE was 15.15% with standard error 3.57% in New York State, and the MAPE was 2.07% with standard error 0.42% in Ohio.**

| New York |  |  |  |  |  |  |  |  |  |  |  |  |  |  |
| --- | --- | --- | --- | --- | --- | --- | --- | --- | --- | --- | --- | --- | --- | --- |
| Date | 09/01 | 09/02 | 09/03 | 09/04 | 09/05 | 09/06 | 09/07 | 09/08 | 09/09 | 09/10 | 09/11 | 09/12 | 09/13 | 09/14 |
| Observed | 432 | 445 | 430 | 428 | 425 | 410 | 413 | 445 | 463 | 482 | 473 | 467 | 464 | 464 |
| Predicted | 584 | 569 | 555 | 541 | 529 | 517 | 507 | 498 | 489 | 481 | 473 | 466 | 461 | 456 |
| Ohio |  |  |  |  |  |  |  |  |  |  |  |  |  |  |
| Date | 09/01 | 09/02 | 09/03 | 09/04 | 09/05 | 09/06 | 09/07 | 09/08 | 09/09 | 09/10 | 09/11 | 09/12 | 09/13 | 09/14 |
| Observed | 777 | 746 | 742 | 727 | 717 | 691 | 686 | 687 | 700 | 682 | 650 | 626 | 635 | 671 |
| Predicted | 771 | 760 | 748 | 737 | 725 | 714 | 703 | 692 | 681 | 671 | 660 | 649 | 639 | 629 |

1. **The interface of the online R shiny app.**

**Figure S5: The interface of the online R shiny app. The app is available at:** [**https://riskcalc.org/DSEIR/**](https://riskcalc.org/DSEIR/)**.**


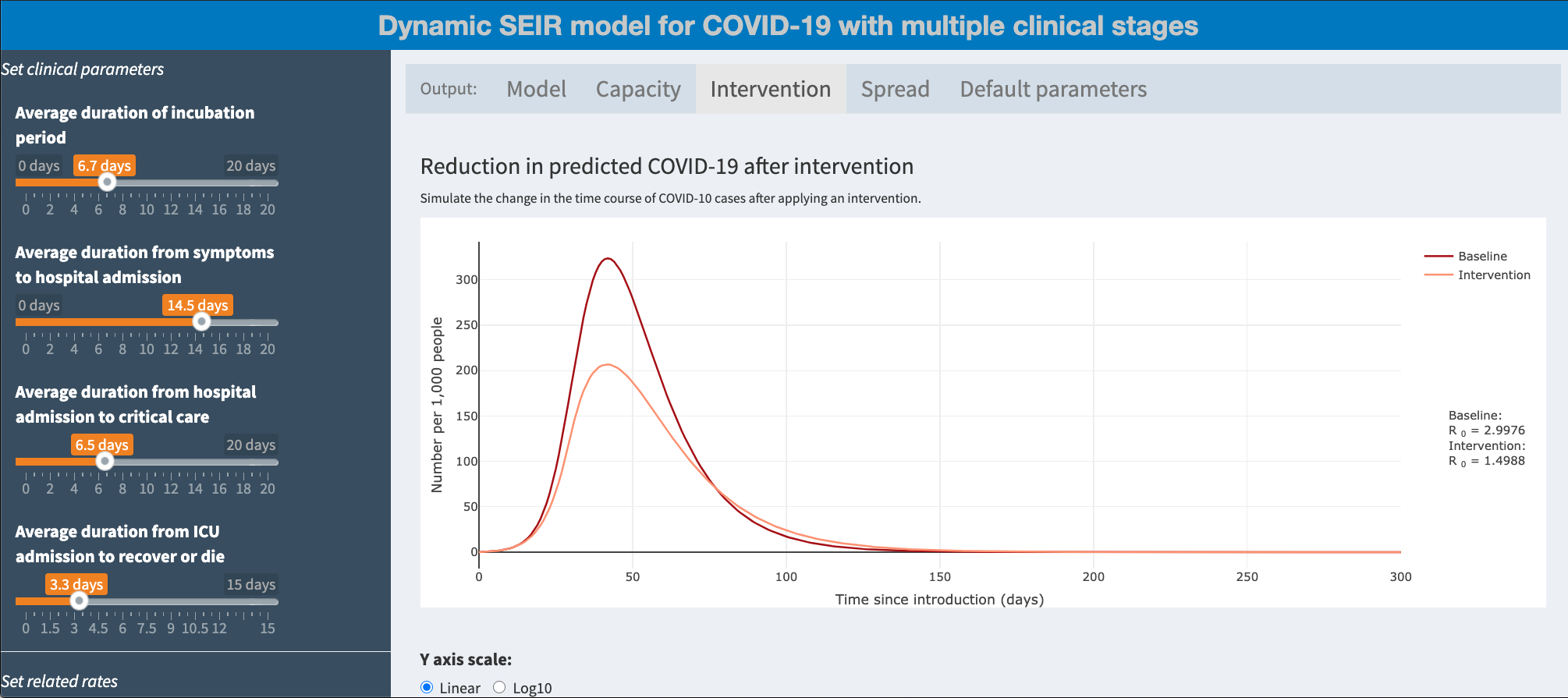

Supplement: Multimedia Appendix 1 [file publichealth_v6i4e25174_app1.docx]
